# Successful ventilator weaning after aerosolized liposomal amphotericin B in persistent *Candida glabrata* colonization: a case report

**DOI:** 10.3389/fmed.2025.1697842

**Published:** 2025-12-04

**Authors:** Massimo Caracciolo, Simona Pellicano, Sarah Antonella Caracciolo, Maria Francesca Stagno, Antonino Ripepi, Selma Valerie Mammone, Nadia Pellicano, Stefano La Scala, Giuseppe Mazza, Giuseppe Neri

**Affiliations:** 1 UOSD Post-Operative Intensive Care Unit, Great Metropolitan Hospital Bianchi Melacrino Morelli, Reggio Calabria, Italy; 2 UOC Perioperative and General Anesthesia and Intensive Care, Senese University Hospital, Siena, Italy; 3 UOC Anesthesia and Intensive Care, San Pio X Hospital Humanitas, Milan, Italy; 4 Anesthesia and Intensive Care, Department of Medical and Surgical Sciences, Magna Grecia University, Catanzaro, Italy

**Keywords:** aerosolized amphotericin B, *Candida glabrata Nakaseomyces glabrata*, ventilator weaning, ICU, liposomal formulation, Aerogen vibrating mesh device, immune evasion, fungal colonization

## Abstract

**Background:**

Invasive candidiasis and persistent fungal colonization of the airways are increasingly recognized as potential barriers to successful weaning from mechanical ventilation in critically ill patients. Among *non-albicans Candida species*, *Candida glabrata*, recently reclassified as *Nakaseomyces glabrata,* is particularly challenging due to its unique mechanisms of resistance and immune evasion. The use of aerosolized liposomal amphotericin B (Ambisome®) offers a promising local therapeutic approach, combining targeted delivery with a favorable safety profile.

**Case presentation:**

We present the case of a 68-year-old man who required prolonged mechanical ventilation following an ischemic stroke and subsequently developed persistent bronchial colonization by *Candida glabrata*. Despite systemic antifungal therapy, the colonization persisted, and pulmonary compliance remained poor. The administration of aerosolized liposomal amphotericin B (10 mg BID for 10 days) led to significant improvement in respiratory mechanics and allowed for successful weaning from the ventilator. Follow-up cultures confirmed microbiological clearance.

**Conclusion:**

This case highlights the potential role of aerosolized liposomal amphotericin B as an effective and well-tolerated treatment for persistent fungal airway colonization by *C. glabrata*, even in non-immunocompromised ICU patients.

## Introduction

Fungal colonization of the respiratory tract in intensive care patients has traditionally been regarded as clinically irrelevant (1). However, increasing evidence suggests that in certain clinical contexts especially when weaning from mechanical ventilation is delayed this colonization may contribute to clinical deterioration (2). *Candida glabrata*, recently reclassified as *Nakoseomyces glabrata*, unlike its more commonly encountered cousin *Candida albicans*, is gaining attention due to its ability to persist in host tissues and its resistance to standard antifungal therapies.

Several characteristics make *C. glabrata* particularly problematic: it has reduced susceptibility to azole antifungals, readily forms biofilms on mucosal surfaces and medical devices, and employs sophisticated immune evasion strategies (3, 4). In such cases, systemic therapy alone may be insufficient, especially in patients with impaired perfusion or altered pulmonary dynamics.

Aerosolized liposomal amphotericin B provides an alternative route of administration that ensures high local drug concentrations in the lungs while minimizing systemic exposure and toxicity (1). Although data supporting its use have mainly focused on immunocompromised populations, there is growing interest in its application in the broader ICU setting. Recent reviews and case series have begun to explore the utility of inhaled antifungals in patients without classical immunosuppression (5, 6), but robust clinical data remain limited.

## Case presentation

A 68-year-old man, with a past medical history of ischemic cardiomyopathy and COPD, was admitted to our ICU following an acute ischemic stroke. Imaging showed severe stenosis of the right internal carotid artery (ICA) and a large ischemic penumbra in the fronto-parieto-temporal region. Emergency thrombectomy and ICA stenting were successfully performed. His cardiovascular history included an anterior myocardial infarction treated with thrombolysis, followed by coronary stenting of the LAD and circumflex artery. He was a former smoker with COPD and hypertension, without family or genetic disorders. The patient lived with his family and had no psychosocial risk factors.

Initial ICU management included mechanical ventilation, neuroprotection, ulcer and deep vein thrombosis (DVT) prophylaxis, and enteral nutrition. A percutaneous tracheostomy was placed on day 7 due to ongoing ventilatory dependence. At ICU admission, he was hemodynamically stable and afebrile, with normal renal and hepatic function and mild leukocytosis. Neurological evaluation showed left hemiplegia and hemineglect.

Repeated bronchial aspirates yielded *Candida glabrata*, despite systemic antifungal therapy. Urine cultures grew *Pseudomonas aeruginosa* (treated with ceftolozane/tazobactam and gentamicin). Despite stable hemodynamics and neurologic improvement, weaning attempts failed due to low pulmonary compliance and elevated airway pressures. Systemic antifungal therapy with fluconazole, followed by anidulafungin, did not eradicate *C. glabrata*. Persistent colonization and impaired compliance prompted the initiation of aerosolized liposomal amphotericin B.

A 10-day course of aerosolized liposomal amphotericin B (Ambisome®, 10 mg BID via nebulizer) was initiated. The therapy was well tolerated without bronchospasm or renal impairment.

**Figure 1:**
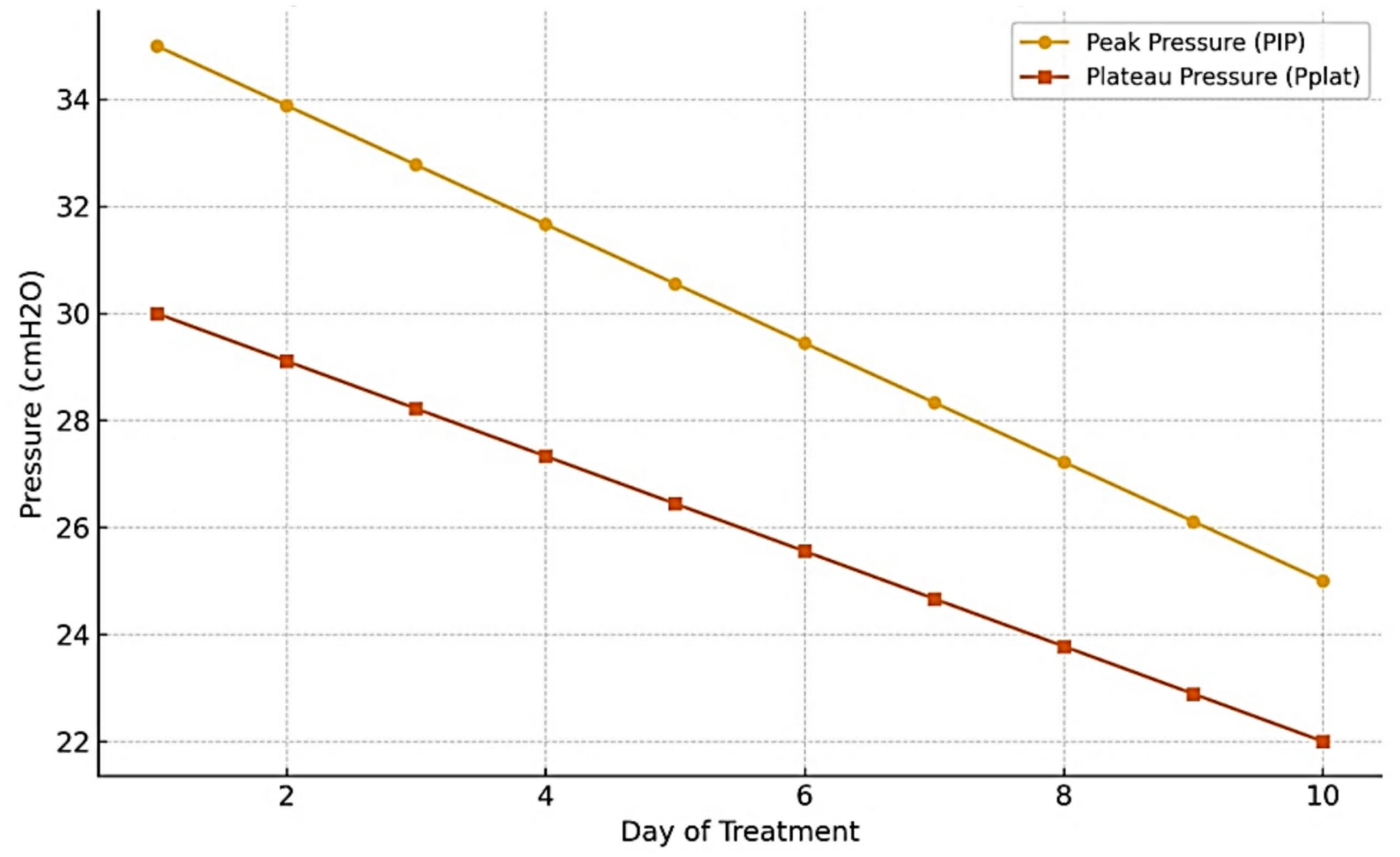
Trend of peak and plateau airway pressures during antifungal therapy.

**Figure 2:**
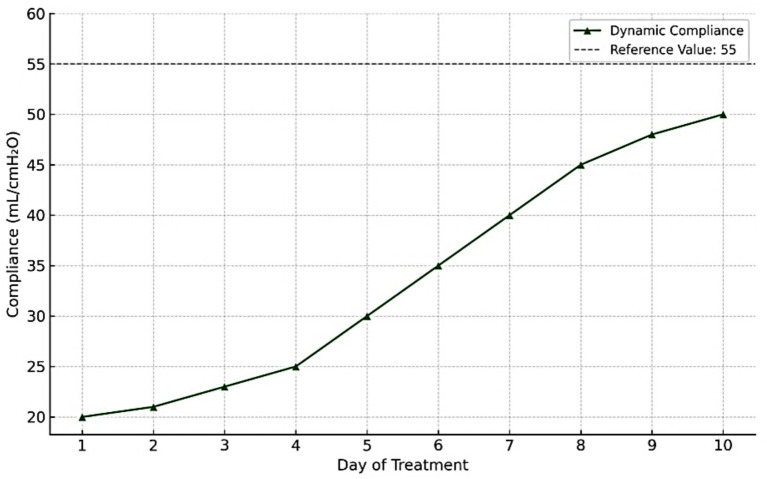
Progressive improvement in dynamic pulmonary compliance.

Clinical response:

- Airway pressures (peak and plateau) gradually decreased (Figure 1).
- Dynamic pulmonary compliance steadily improved (Figure 2).
- Microbiological clearance was confirmed by negative follow-up bronchial cultures.
- The patient was successfully weaned and transferred to neurorehabilitation.

During rehabilitation, the patient progressively regained consciousness and comprehension, though left hemiplegia with mild spasticity and neglect persisted. Respiratory function remained stable after tracheostomy decannulation, which occurred after 38 days, with spontaneous breathing on room air. Oral feeding was restored after swallowing therapy, and the nasogastric tube was removed and urinary catheterization was discontinued. At the time of discharge, which occurred after approximately three months of neurorehabilitation, he was awake, cooperative, hemodynamically stable, and free of fungal or respiratory relapse, though still functionally dependent.

## Discussion

In the last six months at our hospital, *Candida glabrata* accounted for 27.6% of all *Candida* spp. isolates from blood culture samples, ranking as the second most frequently detected species after *Candida parapsilosis*, which represented 51.7% of isolates. This local trend mirrors global data showing an increasing prevalence of non-*albicans Candida* species, emphasizing the clinical importance of *C. glabrata* as an emerging opportunistic pathogen in critically ill patients.

Persistent airway colonization by *Candida glabrata* is not always as clinically irrelevant (1). In select cases, it may contribute to worsening ventilatory function and delayed weaning, even without a diagnosis of invasive fungal infection. Several mechanisms help explain this phenomenon.

**Figure 3:**
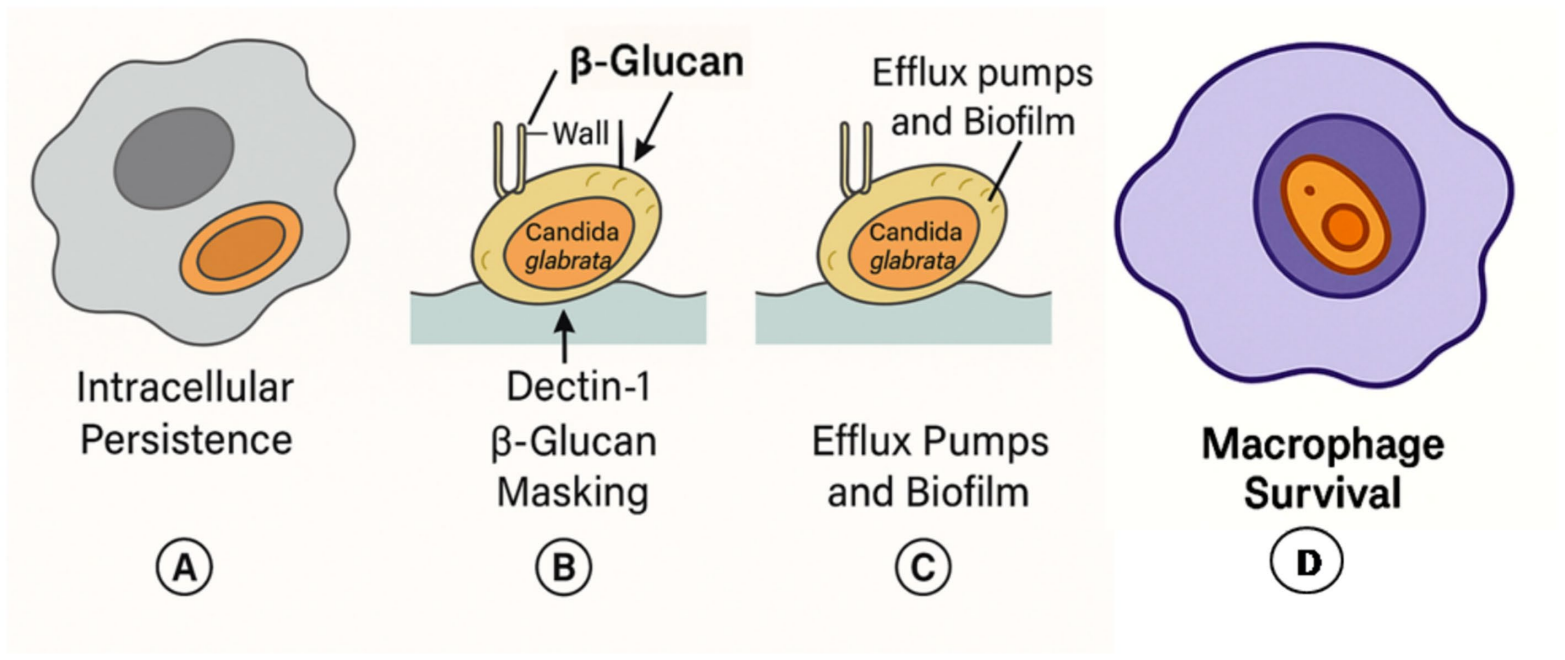
Schematic representation of *Candida glabrata* immune evasion mechanisms: **(A)** intracellular persistence, **(B)** masking of β-glucans to avoid recognition by Dectin-1, **(C)** efflux pump activity and biofilm formation, **(D)** macrophage survival.

*C. glabrata* is adept at avoiding immune detection. Its ability to persist inside macrophages without triggering strong inflammatory responses allows it to remain in host tissues for extended periods. Furthermore, it alters the composition of its cell wall to mask β-glucans from host pattern-recognition receptors such as Dectin-1, reducing immune activation (7, 8) (Figure 3).

Another challenge is the organism’s ability to form resilient biofilms, particularly on endotracheal tubes and ventilator circuits. These biofilms are inherently resistant to antifungal drugs and host defenses, and can lead to localized inflammation, increased secretion viscosity, and partial airway obstruction. These changes translate into higher airway pressures and reduced compliance (4).

Observational studies have linked fungal airway colonization to increases in both peak inspiratory pressure (PIP) and plateau pressure (Pplat). Even in the absence of invasive disease, these findings suggest that colonization itself may compromise respiratory mechanics (1, 9, 10).

Adding to this, *C. glabrata*’s evolutionary success as a pathogen lies in its adaptability and stealth. Unlike more aggressive fungi that trigger overt inflammation, *C. glabrata* survives within the host by flying under the immune radar. It adapts to various environmental stresses, including oxidative bursts, nutrient scarcity, and pH shifts, thanks to highly efficient genetic regulation (11). Key transcription factors, such as CgPdr1, orchestrate resistance to antifungals and modulate genes involved in metabolism and host interaction (3, 12). Genomic plasticity further enhances its survival capacity (10). The organism also activates antioxidant enzymes that neutralize reactive oxygen species generated by immune cells (13). These features allow *C. glabrata* to persist in a quiescent state within the host, contributing to chronic colonization without necessarily causing invasive disease. This stealthy behavior may explain why standard antifungal therapies often fail to eradicate it from the airways (8).

**Figure 4:**
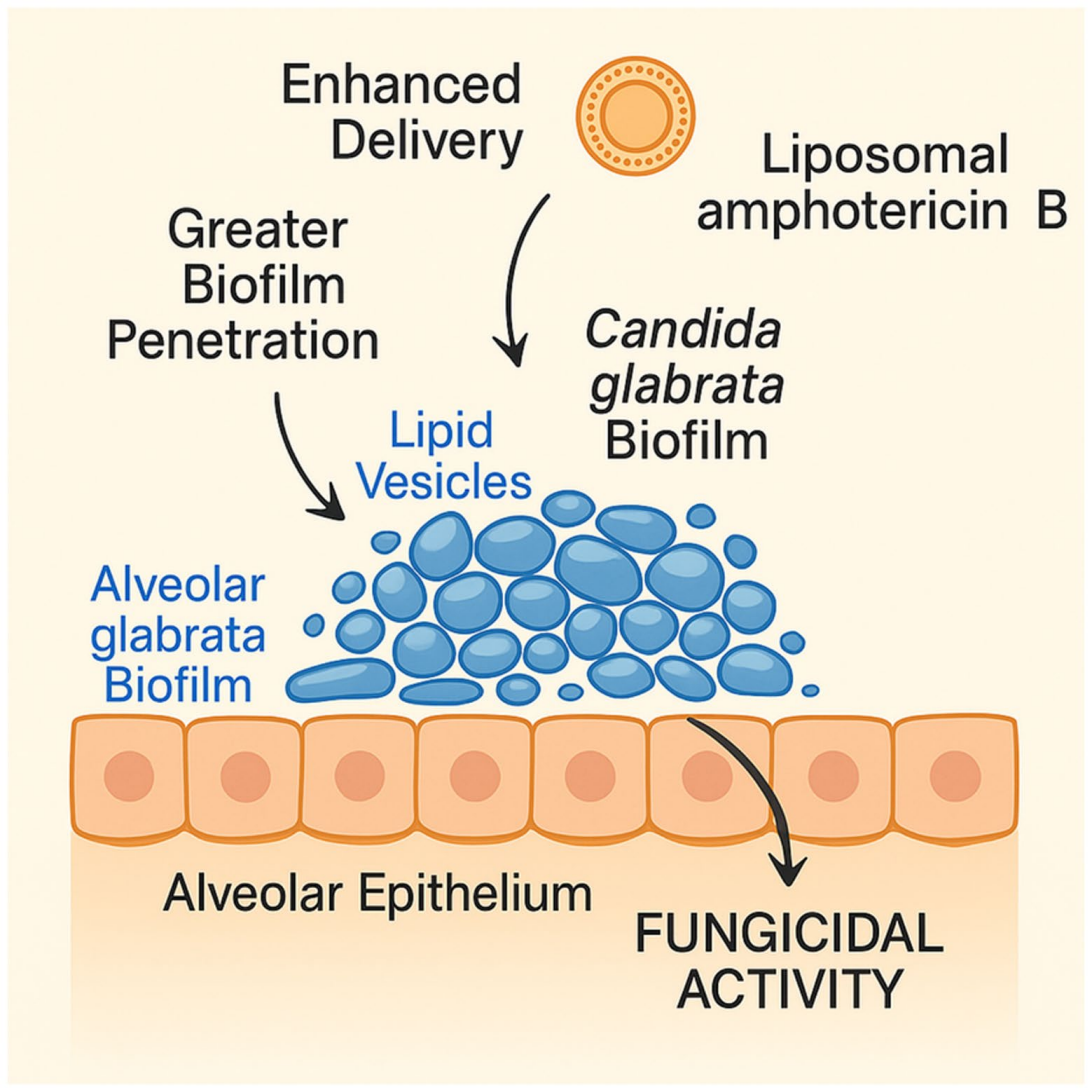
Mechanism of action of aerosolized liposomal amphotericin B: liposomal particles deposit in alveolar spaces, penetrate biofilm matrices, and deliver amphotericin B directly to fungal membranes, achieving high local fungicidal activity with minimal systemic exposure.

The pharmacologic profile of liposomal amphotericin B makes it well-suited for aerosolized delivery. AmBisome® particles, measuring 55 to 75 nm, are designed to achieve efficient tissue penetration and prolonged systemic circulation, reducing toxicity and improving bioavailability (14, 15). The choice of nebulization system is critical. The Aerogen vibrating mesh device produces fine, uniform particles (3–5 μm) without heating the drug, thereby preserving the integrity of the liposomal structure (Figure 4). This ensures effective pulmonary deposition, high local drug concentration, and better tolerability, making it ideal for critically ill patients on ventilators 2020 (16–18).

## Conclusion

In this case, the use of aerosolized liposomal amphotericin B was safe, well tolerated, and clinically effective in a ventilated ICU patient with persistent *Candida glabrata* colonization. The patient’s improvement in respiratory mechanics and successful weaning highlight the therapeutic potential of this targeted approach.

This report adds to the growing body of evidence supporting the use of aerosolized antifungal therapy beyond immunocompromised populations, particularly when persistent colonization compromises ventilatory function (19-22). Further clinical trials and real-world observational data are needed to better define the role of inhaled antifungals in critically ill patients and to standardize protocols for their use.

### Strengths and limitations

This is a single-patient case report, and its findings cannot be generalized to broader populations. Long-term follow-up data are unavailable, limiting the assessment of treatment durability. Nevertheless, the detailed clinical, microbiological, and physiological documentation provides valuable preliminary evidence supporting aerosolized liposomal amphotericin B as a potential adjunctive therapy for persistent fungal airway colonization in non-immunocompromised ICU patients.

## Data Availability

The raw data supporting the conclusions of this article will be made available by the authors, without undue reservation.
